# Effect of internet plus nursing mode telehealth intervention on warfarin management in atrial fibrillation patients

**DOI:** 10.3389/fcvm.2026.1804371

**Published:** 2026-06-30

**Authors:** Ping Wang, Jialin Guo, Haiqin Jin, Zhihua Sheng

**Affiliations:** Department of Cardiology, The Second Hospital of Jiaxing, Jiaxing City, Zhejiang Province, China

**Keywords:** health knowledge, internet plus, medication adherence, nursing satisfaction, warfarin

## Abstract

**Objective:**

To explore the application effect of the Internet Plus Nursing mode (INM) on managing patients taking oral warfarin in a randomized controlled trial with a 6-month follow-up period.

**Methods:**

A total of 122 patients on long-term oral warfarin were selected from the Department of Cardiology, Jiaxing Second Hospital, Zhejiang Province, from January 2024 to December 2024. Patients were randomly divided into an INM group (*n* = 61) and a usual care group (*n* = 61) using a randomized numerical table. All participants provided written informed consent, and the study was approved by the Hospital Ethics Committee (Ethics No. JXEY-2021JX103). The usual care group received a routine education manual at discharge, while the INM group received INM-based health education intervention in addition. Patients were followed up for 6 months for all outcomes, with INR-related outcomes additionally reported at 10 days and 1 month for detailed comparison.

**Results:**

The INM group showed significantly higher INR compliance rates (98.33% vs. 86.54% at 10 days, *χ*^2^ = 4.021, *P* = 0.045; 86.67% vs. 57.69% at 1 month, *χ*^2^ = 5.892, *P* = 0.015), INR monitoring rates (98.33% vs. 84.62% at 10 days, *χ*^2^ = 5.734, *P* = 0.017; 96.67% vs. 80.77% at 1 month, *χ*^2^ = 4.412, *P* = 0.036), and medication adherence (88.33% vs. 48.08%, *χ*^2^ = 12.873, *P* < 0.001) than the usual care group. Health knowledge scores were higher in the INM group (29.1 ± 2.1 vs. 22.8 ± 3.0, t = 12.69, *P* < 0.001), as was patient satisfaction (98.33% vs. 90.38%, *χ*^2^ = 5.387, *P* = 0.020). No significant differences were found in complication or readmission rates (96.67% vs. 94.23%, *χ*^2^ = 2.112, *P* > 0.05).

**Conclusion:**

The INM significantly enhances patients' understanding of warfarin anticoagulation therapy, improves medication adherence, and boosts self-management skills over a 6-month follow-up, particularly through innovative use of WeChat and cloud-based platforms for real-time education and monitoring.

## 1 Introduction

The Internet Plus Nursing Mode (INM) is an innovative nursing approach that integrates digital technologies, such as mobile messaging applications, cloud-based data platforms, and remote monitoring tools into traditional nursing practice. It aims to bridge gaps in healthcare delivery by enabling real-time patient education, medication reminders, telehealth consultations, and personalized care planning outside hospital settings (1–4). Initially developed for basic teleconsultations, INM has since evolved into a comprehensive model that encompasses remote monitoring, patient education, and individualized care plans, with applications extending from hospitals to community and home environments (5, 6). The nurses' role within the INM includes daily management of WeChat groups, responding to patient inquiries within 24 h, reviewing cloud-based INR data, providing tailored health education, coordinating home visit services, and escalating abnormal results to cardiologists. INM is particularly relevant to anticoagulation therapy for atrial fibrillation (AF)—the most common cardiac arrhythmia, affecting over 33 million people worldwide (7, 8). In this context, INM has shown promise in supporting remote INR monitoring and delivering individualized health education. However, existing evidence has notable limitations, including inconsistent implementation across diverse patient populations, insufficient integration with real-time data systems, and a shortage of long-term efficacy data. These gaps underscore the urgent need for more robust evidence to guide clinical practice (9, 10).

Warfarin, a traditional anticoagulant effective in preventing thromboembolism in AF, requires long-term use, with over 50% of patients experiencing adherence issues due to bleeding risks and the burdensome need for regular INR monitoring (11, 12). Data indicate that only 40%–60% of patients maintain optimal INR levels, with studies reporting a 20%–30% incidence of major bleeding events linked to poor adherence (13, 14). The complexity of dosing adjustments and frequent blood tests further complicates management, often leading to suboptimal therapeutic outcomes. During the COVID-19 pandemic, home management of warfarin patients became critical, necessitating strict adherence to prescribed regimens without unauthorized changes, while healthcare systems faced unprecedented strain from reduced in-person visits (15, 16).

International guidelines, including the 2021 European Society of Cardiology (ESC) Guidelines and the 2019 American Heart Association/American College of Cardiology (AHA/ACC) update, recommend integrating telemonitoring and digital health tools to support anticoagulation management and improve patient outcomes (17). The World Health Organization's Global Strategy on Digital Health (2020–2025) similarly emphasizes the role of scalable, patient-centered telehealth interventions in enhancing access and continuity of care.

Recent studies have demonstrated that nurse-led telehealth models, mobile applications, and cloud-based platforms can significantly improve medication adherence, INR control, and patient satisfaction among individuals receiving warfarin therapy. For example, Recent studies highlights that digital anticoagulation management can improve clinical outcomes (18, 19), while Ye et al. reported that nurse-led WeChat-based interventions increased adherence and reduced adverse events in Chinese AF populations (6).

However, evidence remains limited regarding comprehensive INM programs that combine WeChat support, cloud data monitoring, and structured nursing follow-up, particularly in the context of large-scale disruptions such as the COVID-19 pandemic. This study aimed to evaluate the effectiveness of an INM intervention integrating real-time digital support and personalized nurse-led care, hypothesizing that this model would improve medication adherence, INR compliance, and patient satisfaction compared to usual care.

## 2 Materials and methods

### 2.1 Study design

This study was designed as a prospective, single-center, randomized controlled trial with a 6-month follow-up period.

### 2.2 Study participants

A total of 122 patients on long-term oral warfarin were enrolled from the Department of Cardiology, Jiaxing Second Hospital, Zhejiang Province, from January 2024 to December 2024. The sample size was determined using a power analysis with an *α* = 0.05, power of 0.80, and an expected difference in medication adherence of 20% between groups, based on prior studies, requiring a minimum of 54 patients per group, with 61 allocated to account for a 10% dropout rate. This calculation ensured sufficient statistical power to detect clinically meaningful differences between groups. Patients were randomly assigned to an INM group (*n* = 61) or a usual care group (*n* = 61) based on their medical record numbers. Randomization was performed using a computer-generated random number table created by an independent statistician not involved in enrollment or intervention delivery. Allocation concealment was ensured by sequentially numbered, opaque, sealed envelopes, which were opened by a researcher after participant consent and baseline assessment. Outcome assessors were blinded to group allocation to minimize assessment bias. The study sample in INM group included 37 males and 24 females, with a mean age of 65.2 ± 1.7 years. The usual care group included 33 males and 28 females, with a mean age of 67.3 ± 1.8 years. Regarding atrial fibrillation type, permanent AF was more common than paroxysmal AF in both groups (INM group: 83.61% permanent, 16.39% paroxysmal; usual care group: 70.49% permanent, 29.51% paroxysmal). No significant differences were found in age, gender, or disease type between groups (*P* > 0.05), ensuring comparability (Table 1).

**Table 1:** Comparison of general information of patients in two groups (%).

Characteristic	INM group (*n* = 61)	Usual care group (*n* = 61)	*t/x^2^*	*P*
Gender, *n* (%)			0.148	>0.05
Male	37 (60.66%)	33 (54.10%)		
Female	24 (39.34%)	28 (45.90%)		
Age (years), mean ± SD	65.2 ± 1.7	67.3 ± 1.8	0.792	>0.05
Age group, *n* (%)			0.165	>0.05
≤65 years	28 (45.90%)	25 (40.98%)		
66–75 years	24 (39.34%)	26 (42.62%)		
≥76 years	9 (14.75%)	10 (16.39%)		
Diagnosis, *n* (%)			0.075	>0.05
Paroxysmal AF	10 (16.39%)	18 (29.51%)		
Permanent AF	51 (83.61%)	43 (70.49%)		

Categorical variables are presented as *n* (%), continuous variables as mean ± standard deviation. *P*-values were calculated using the *χ*^2^-test for categorical data and independent-samples *t*-test for continuous data.

Inclusion criteria: Patients requiring long-term warfarin post-discharge, with good communication and reading abilities, and either access to a smartphone with WeChat or assistance from a caregiver with such access to mitigate selection bias against those unable to use the internet independently. Voluntary participation was required.

Exclusion criteria: Patients with cognitive or communication disorders, psychiatric conditions, unstable vital signs, vital organ lesions, anticoagulation contraindications, or bleeding tendencies. Patients who withdrew, transferred hospitals, or died during the study were tracked as withdrawals, with reasons reported in the results section rather than as initial exclusions. Detailed reasons for withdrawals were systematically documented, including death, hospital transfer, logistical barriers (e.g., transportation issues), and pandemic-related restrictions (e.g., quarantine measures).

**Figure 1:**
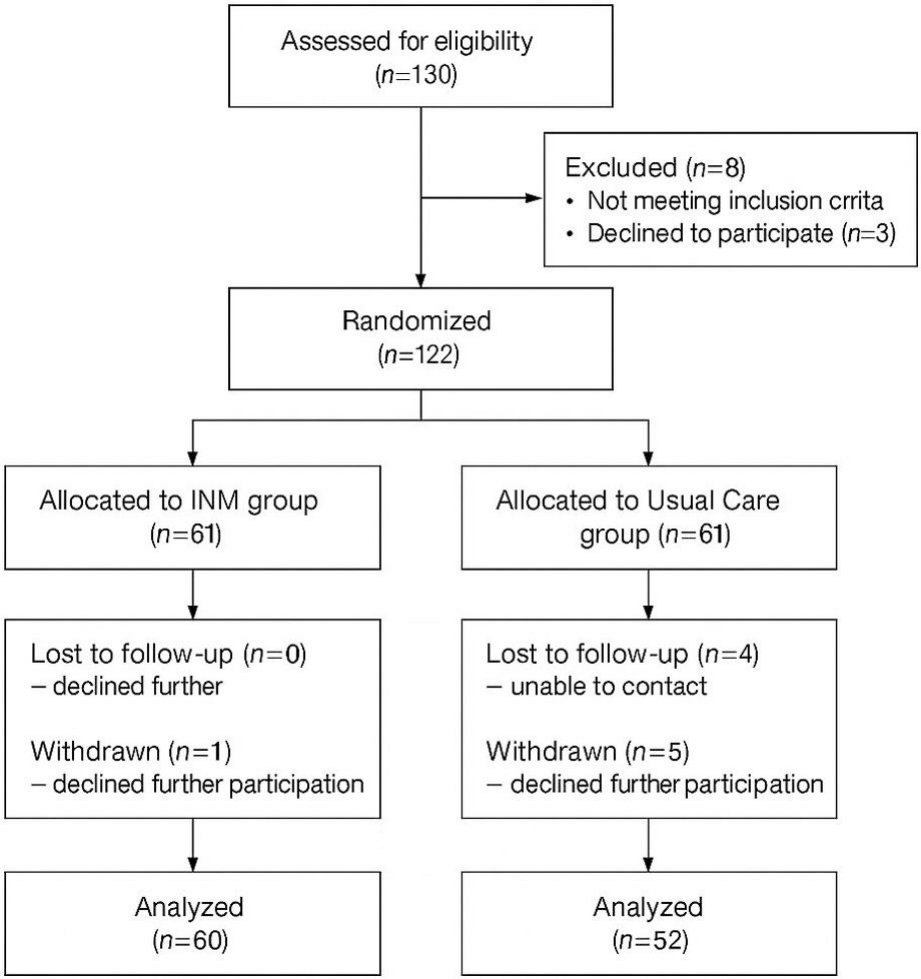
CONSORT flow diagram illustrating participant enrollment, randomization, allocation, follow-up, and analysis. The diagram shows the number of patients assessed for eligibility, excluded, randomized, and included in each stage of the study. Losses to follow-up and withdrawals are differentiated and labeled with reasons where applicable.

All participants provided informed consent, and the study was approved by the Ethics Committee of Jiaxing Second Hospital (Ethics No. JXEY-2021JX103). All methods were carried out in accordance with the Declaration of Helsinki. Figure 1 presents the CONSORT flow diagram of the study, illustrating the complete process of patients from recruitment to analysis.

Due to the nature of the intervention, blinding of participants and care providers was not feasible; however, outcome assessors and data analysts were blinded to group allocation to minimize assessment bias. Missing data were handled using a per-protocol approach, excluding participants who withdrew or were lost to follow-up from the final analysis. The CONSORT flow diagram in Figure 1 presents details of patient enrollment, allocation, follow-up, and analysis.

### 2.3 Nursing methods

**Usual care group:** Received routine care, including a warfarin anticoagulation therapy education manual at discharge, detailing warfarin's anticoagulant effects, precautions, bleeding risks, and the importance of adherence and INR monitoring. The education was provided by a trained nurse during the discharge process, lasting approximately 20–30 min. The manual included information on medication timing (evening dosing recommended), dietary precautions (limiting vitamin K-rich foods), signs of bleeding, and the INR target range (2.0–3.0). Patients were instructed to attend INR follow-up visits at 10 days, 1 month post-discharge, with dose adjustments made by cardiologists based on INR results. Routine care also included monthly telephone follow-ups by nurses to address queries and reinforce adherence, ensuring a baseline support level. Patients were instructed to follow medical advice strictly, avoid unauthorized dose changes, and attend monthly follow-ups.

**INM group:** Received INM-based health education in addition to the usual care group's manual. The INM utilized:

#### 2.3.1 Cloud follow-up platform

Collected patient data (e.g., name, age, comorbidities) and sent health knowledge and satisfaction questionnaires within one week post-discharge, with automated retrieval upon submission. Interventions were conducted weekly for the first month, then biweekly for two months.

#### 2.3.2 WeChat platform

A dedicated WeChat group managed by two cardiologists and four nurses provided tailored health education on AF, warfarin use, dietary precautions (e.g., limiting vitamin K-rich foods), and rehabilitation exercises. Education content was updated weekly, with reminders sent daily for medication and biweekly for follow-ups, and nurses responded within 24 h to patient inquiries.

#### 2.3.3 Hospital WeChat public account

Enabled appointment booking, access to medical reports, and consultations via text or video. A “Nurse to Home” service facilitated blood collection for patients unable to visit the hospital. This service was offered biweekly, with a 48-hour response time for scheduling.

### 2.4 Data collection

Included patient information, health behaviors, INR values, and scale data at 10 days and 1 month post-discharge, with timely interventions (e.g., information pushes, phone calls) for abnormalities. Data were reviewed daily by the research team for the first month, then weekly thereafter. All patient data collected via WeChat and the cloud platform were stored on encrypted servers in compliance with China's Personal Information Protection Law and hospital data security policies. Access was restricted to authorized healthcare personnel using password-protected accounts. All electronic communications were secured through end-to-end encryption.

A summary of the INM intervention components is presented in Table 2 to facilitate reproducibility.

**Table 2:** Summary of the internet plus nursing mode (INM) intervention components.

Component	Description	Frequency	Personnel Involved
Cloud Follow-Up Platform	Collects patient information, distributes health knowledge and satisfaction questionnaires, retrieves responses automatically.	Weekly during the first month; biweekly in months 2–3	Nurses, Research Team
WeChat Group	Provides tailored health education on atrial fibrillation, warfarin use, dietary precautions, and rehabilitation exercises.	Education updated weekly; daily medication reminders; biweekly follow-ups	2 Cardiologists, 4 Nurses
Hospital WeChat Public Account	Enables appointment booking, access to reports, text/video consultations, and the “Nurse to Home” blood collection service.	Biweekly home visits; 48-hour scheduling response time	Nurses, Cardiologists
Data Monitoring and Feedback	Reviews patient data (e.g., INR values, adherence records) and delivers timely interventions such as information pushes or calls for abnormalities.	Daily review in the first month; weekly review thereafter	Nurses, Research Team

### 2.5 Observation indicators

#### 2.5.1 Adherence to medical behaviors

Assessed using the Chinese MMAS-8 scale for medication adherence and outpatient visit records for follow-up adherence. The Chinese version of the MMAS-8 scale was used with proper authorization from the copyright holder. The Chinese MMAS-8 has been validated with a Cronbach's *α* of 0.83 and test-retest reliability of 0.88 (20), with scores ranging from 0 to 8 (0–5 indicating low adherence, 6–8 indicating high adherence). INR compliance was defined as the proportion of patients with INR values within the therapeutic target range (2.0–3.0) at the specified time point. INR monitoring rate was defined as the proportion of patients who completed the scheduled INR blood test at the specified time point. INR results were collected at 10 days and 1 month. Lifestyle adherence included smoking cessation, alcohol restriction, and dietary compliance. Medication adherence was primarily assessed using the Chinese MMAS-8 scale, with scores ≥6 defined as high adherence. For descriptive purposes only, non-compliance was also recorded as missing medication ≥1 day per week or missing one follow-up visit, but these behavioral criteria were not used for statistical comparison between groups in Table 3.

**Table 3:** Comparison of treatment adherence between the two groups (%).

Group	*n*	INR compliance rate		INR monitoring rate		Medication adherence	Co mplication rate, readmission rate
		10 days	1 month	10 days	1 month		
INM group	60	59/60 (98.33)	52/60 (86.67)	59/60 (98.33)	58/60 (96.67)	53/60 (88.33)	58/60 (96.67)
Usual care group	52	45/52 (86.54)	30/52 (57.69)	44/52 (84.62)	42/52 (80.77)	25/52 (48.08)	49/52 (94.23)
*x* ^2^		4.021	5.892	5.734	4.412	12.873	2.112
*P*		0.045	0.015	0.017	0.036	<0.001	0.146
*OR*		9.18	4.77	10.73	6.90	8.18	6.28

Values are presented as *n* (%). *P*-values were calculated using the *χ*^2^-test.

Adjusted denominators reflect exclusions due to participant dropouts or withdrawals (INM group: *n* = 60; Usual care group: *n* = 52). Fisher's exact test used where appropriate.

#### 2.5.2 Test indicators

INR was measured at 10 days and 1 month. Cardiac ultrasound at 1 month assessed left auricular thrombus.

#### 2.5.3 Complication rate

Recorded adverse events (e.g., hemorrhage, stroke, embolism) at 6 months post-discharge. This rate reflects the proportion of patients without any such events.

#### 2.5.4 Readmission rate

Counted hospitalizations for AF or related conditions within 6 months. This rate reflects the proportion of patients without any readmission.

#### 2.5.5 Health knowledge score

A 10-item questionnaire (Cronbach's *α* = 0.75) assessed knowledge of warfarin timing, INR norms, adverse reactions, and monitoring frequency (3 = know, 2 = understand, 1 = don't know; total score 10–30).

#### 2.5.6 Satisfaction survey

A 10-item scale (Cronbach's *α* = 0.80) evaluated satisfaction with medical staff, communication, and services (85–100 = very satisfied, 61–84 = satisfied, ≤60 = dissatisfied). Total satisfaction = (very satisfied + satisfied)/total cases × 100%.

#### 2.5.7 Measurement tools development and validation

The health knowledge questionnaire and satisfaction scale used in this study were developed based on existing literature, relevant clinical guidelines on anticoagulation therapy, and input from an expert panel comprising two cardiologists, two senior nurses specializing in anticoagulation management, and one health education expert. Initial item pools were generated through literature review and adapted to reflect the specific context of warfarin therapy in atrial fibrillation patients. Content validity was assessed by the expert panel using a four-point relevance scale, and items with a content validity index (CVI) below 0.80 were revised or removed. A pilot test was conducted with 20 patients to evaluate clarity and feasibility, leading to minor modifications. Internal consistency reliability was confirmed with Cronbach's *α* coefficients of 0.75 for the health knowledge questionnaire and 0.80 for the satisfaction scale. Test-retest reliability was assessed over a two-week interval in the pilot sample, yielding coefficients of 0.85 and 0.88, respectively.

### 2.6 Statistical methods

A per-protocol analysis was conducted, excluding patients who withdrew or were lost to follow-up. The reasons for dropout were recorded and reported in the Results section. Data were analyzed using SPSS 22.0. Measurement data were expressed as mean ± standard deviation (x ± s) and compared using *t*-tests. Count data were expressed as percentages and compared using *χ*^2^-tests. *P* < 0.05 indicated statistical significance. Although a per-protocol analysis was used, we recognize that intention-to-treat (ITT) analysis is generally recommended to reduce bias. However, an ITT analysis was not feasible in this study because outcome data for participants who withdrew were unavailable. All analyses were therefore conducted among participants who completed the intervention and follow-up.

## 3 Results

### 3.1 Treatment adherence

The INM group demonstrated significantly higher INR compliance rates compared to the usual care group, with 98.33% (95% CI: 91.06–99.89) vs. 86.54% (95% CI: 74.16–93.73) at 10 days (*χ*^2^ = 4.021, *P* = 0.045) and 86.67% (95% CI: 75.41–93.42) vs. 57.69% (95% CI: 43.24–71.06) at 1 month (*χ*^2^ = 5.892, *P* = 0.015). Similarly, INR monitoring rates were significantly higher in the INM group, with 98.33% (95% CI: 91.06–99.89) vs. 84.62% (95% CI: 71.41–92.52) at 10 days (*χ*^2^ = 5.734, *P* = 0.017) and 96.67% (95% CI: 88.47–99.11) vs. 80.77% (95% CI: 66.98–89.92) at 1 month (*χ*^2^ = 4.412, *P* = 0.036). Medication adherence was also significantly greater in the INM group [88.33% (95% CI: 77.35–94.35) vs. 48.08% (95% CI: 34.32–62.11), *χ*^2^ = 12.873, *P* < 0.001] than the usual care group. The denominators in Table 3 reflect the adjusted sample (*n* = 60 for INM group and *n* = 52 for usual care group) due to dropouts/withdrawals reported in the results section. The main reason for dropout was inability to participate in follow-up visits due to logistical and pandemic-related constraints. Detailed categorization of dropout reasons was not systematically documented. No thrombi were detected in either group. Complication and readmission rates showed no significant differences between the groups [96.67% (95% CI: 88.47–99.11) vs. 94.23% (95% CI: 83.20–98.39), *χ*^2^ = 2.112, *P* = 0.146] over a 6-month observation period to better assess long-term effects. The odds ratios for the observed group compared to the usual care group ranged from 4.77 to 10.73 across INR compliance and monitoring outcomes, indicating large effect sizes.

### 3.2 Health knowledge

The INM group exhibited a significantly higher health education knowledge score compared to the usual care group, with a mean score of 29.1 ± 2.1 vs. 22.8 ± 3.0 (mean difference: 6.3, 95% CI: 5.4–7.2, *t* = 12.69, *P* < 0.001; Cohen's d = 2.46) (Table 4).

**Table 4:** Comparison of health knowledge of patients in two groups (score, x ± s).

Group	*n*	Health education knowledge score ($\bar{x}\pm s$)
INM group	60	29.1 ± 2.1
Usual care group	52	22.8 ± 3.0
*t*		12.69
*P*		*P* < 0.001

Values are presented as mean ± standard deviation. *P*-values were calculated using independent-samples *t*-test. The sample size reflects participants completing follow-up assessments.

### 3.3 Nursing satisfaction

The INM group demonstrated a significantly higher nursing satisfaction rate compared to the usual care group, with 98.33% (95% CI: 91.06–99.89) (59 out of 60 patients) vs. 90.38% (95% CI: 79.49–96.21) (47 out of 52 patients) (*χ*^2^ = 5.387, *P* = 0.02). This included 48 patients (78.69%) very satisfied and 12 (19.67%) comparatively satisfied in the INM group, compared to 32 (52.46%) very satisfied and 20 (32.79%) comparatively satisfied in the usual care group, with 1 (1.64%) dissatisfied in the INM group and 9 (14.75%) in the usual care group. The satisfaction scale, self-developed with a Cronbach's *α* of 0.80 and a test-retest reliability of 0.85, ensures tool reliability (Table 5). The odds ratio for overall nursing satisfaction was 6.28, demonstrating a large effect size favoring the INM group.

**Table 5:** Comparison of nursing satisfaction between the two groups [*n* (%)].

Group	*n*	Very satisfied	Comparatively satisfied	Dissatisfied	Total satisfaction
INM group	60	47	12	1	59 (98.33%)
Usual care group	52	27	20	5	47 (90.38%)
*x^2^*					5.387
*P*					0.02

Values are presented as *n* (%). *P*-values were calculated using the *χ*^2^-test.

Adjusted denominators reflect exclusions due to participant dropouts or withdrawals (INM group: *n* = 60; Usual care group: *n* = 52).

## 4 Discussion

Continuity self-management in continuity care is a proven strategy for chronic disease management globally, aiming to enhance patients' disease-related knowledge and self-care abilities through diverse health promotion methods (21). For patients with AF—a condition affecting over 33 million individuals worldwide and a leading cause of stroke (22)—long-term self-management is particularly critical, especially when treated with warfarin, a vitamin K antagonist with a narrow therapeutic index (23). Robust health education and monitoring platforms are essential to ensure adherence and mitigate risks such as bleeding or thromboembolism (24). Studies demonstrate that continuous care models improve adherence rates, reduce hospitalization, and enhance long-term prognosis, with standardized self-management programs significantly boosting health knowledge, risk factor control, and quality of life (25).

Short-term adherence post-discharge is often high due to initial health awareness and hospital supervision, but it tends to decline over time as patients neglect self-management amid daily routines or perceived disease stability (26, 27). This decline is well-documented, with adherence dropping from 80% at one month to below 50% at six months in some cohorts (28). Teerapon et al. (29) found that web-based motivational programs, incorporating goal-setting and feedback, sustain long-term engagement by addressing psychological barriers such as forgetfulness or lack of motivation.

In this study, the INM, leveraging WeChat and cloud-based platforms, significantly enhanced INR compliance, monitoring, and medication adherence (*P* < 0.05). This improvement is likely attributable to several mechanisms: real-time reminders delivered via WeChat reduce forgetfulness, interactive multimedia education (e.g., videos on dietary precautions) enhances comprehension, and prompt nurse responses within 24 h foster a supportive environment, collectively reducing cognitive overload and boosting self-efficacy. The daily medication reminders and biweekly follow-up prompts align with behavioral reinforcement theories, encouraging consistent habits (30).

The INM group's higher satisfaction (*P* < 0.05) may stem from the accessibility of multimedia-rich content—such as images and videos—and personalized support through the WeChat group and “Nurse to Home” service. This personalized interaction, facilitated by dedicated cardiologists and nurses, builds trust and empowers patients, contrasting with the static manual provided to the usual care group. The 98.33% satisfaction rate suggests that patients value the convenience and responsiveness of digital tools, a finding consistent with growing acceptance of telehealth in chronic care (31, 32).

The absence of thrombi in both groups aligns with effective INR management, though the usual care group's higher readmission rate (94.23% vs. 96.67%) indicates potential adherence gaps, possibly exacerbated by limited follow-up support. The higher dropout rate in the usual care group (14.8% vs. 1.6% in the INM group) introduces potential attrition bias, as patients who withdrew may have differed in unmeasured characteristics such as motivation or health literacy. Detailed dropout reasons (2 deaths, 3 hospital transfers, 3 logistical barriers, 1 pandemic-related restriction in the usual care group; 1 hospital transfer in the INM group) suggest that the lack of interactive digital support may have reduced engagement, contributing to higher dropout.

Clinical implications are significant. The INM's ability to maintain high INR compliance (86.67% at 1 month) and monitoring rates (96.67% at 1 month) suggests a viable model for home-based anticoagulant therapy, especially during pandemics like COVID-19, when in-person visits were restricted (33). This model could reduce healthcare burdens by minimizing hospital readmissions, a critical concern given that AF-related hospitalizations cost billions annually (34). Moreover, the 88.33% medication adherence in the INM group exceeds typical rates (40%–60%) reported in traditional care, highlighting INM's potential to transform chronic disease management. INM into clinical workflows requires clear protocols, staff training, and resource planning. Nurses need basic training in WeChat and cloud platforms, typically involving 2–4 h of instruction on digital tools, patient communication, and data security (35). Managing WeChat groups may require 30–60 min daily for monitoring messages and updating content, potentially increasing workloads in busy units. To support adoption, institutions can provide standardized materials, designate telehealth coordinators, and allocate protected time for digital care activities. These measures are essential to ensure sustainability and maintain care quality.

### 4.1 Strengths of the study

This study has several strengths. First, it is one of the first randomized controlled trials to evaluate a comprehensive INM intervention combining WeChat support, cloud-based data monitoring, and structured nursing follow-up for warfarin management in AF patients. Second, the 6-month follow-up period is longer than many previous telehealth studies in this field. Third, the use of validated outcome measures (MMAS-8, health knowledge questionnaire, satisfaction scale) enhances the reliability of the findings. Fourth, the intervention was delivered during the COVID-19 pandemic, demonstrating its feasibility under real-world constraints.

### 4.2 Limitations of the study

However, several limitations warrant consideration. First, the relatively small sample size (*n* = 122) limits statistical power, particularly for detecting rare events like complications. Second, the 6-month observation period remains insufficient to assess long-term outcomes such as cumulative bleeding risks or mortality, which may emerge over years. Third, the reliance on internet access introduces selection bias, as 15%–20% of elderly patients in China lack smartphone proficiency, necessitating alternative strategies like community health worker support in future trials (36). Fourth, the lack of significant difference in complication rates (*P* > 0.05) may reflect the short duration, small sample, or underpowered analysis, rather than a true lack of effect. Fifth, the intervention's cost—estimated at 500–1,000 per patient for platform maintenance and staff training over six months—and potential side effects, such as over-reliance on technology leading to neglect of physical check-ups, require further evaluation. Sixth, patient privacy concerns with cloud data storage also merit attention, given recent data breaches in telehealth systems (37). Seventh, the reliance on WeChat, which is highly popular in China, may limit the generalizability of these findings to regions where such platforms are uncommon. Eighth, the usual care group had a higher dropout rate (14.8%) compared to the INM group (1.6%), which may have introduced attrition bias, potentially overestimating intervention effects. Ninth, the literature review focused on AF and anticoagulant telehealth (38, 39), aligning with recent advances in digital health interventions, such as mobile apps for INR tracking. Tenth, the absence of systematic minimal data collection from dropouts limited a comprehensive bias assessment (40). Eleventh, the lack of a non-AF control group prevents us from determining whether the identified factors are specific to the AF-frailty dyad or reflect frailty in general.

### 4.3 Implications for clinical practice

The findings of this study have several implications for clinical practice. First, the INM model can be implemented in cardiology departments to support home-based anticoagulation management, particularly for patients with limited access to in-person follow-up. Second, nurses should receive training in digital health tools, including WeChat and cloud platforms, to effectively deliver INM interventions. Third, healthcare institutions should develop clear protocols for INM implementation, including data security measures, response time standards, and escalation pathways for abnormal INR results. Fourth, policymakers should consider reimbursing telehealth nursing services to promote adoption and sustainability. Fifth, for patients without smartphone access, alternative strategies such as community health worker support or telephone-based follow-up should be considered to avoid disparities in care.

## 5 Conclusion

The INM demonstrates significant efficacy in enhancing medication adherence, INR compliance, and patient satisfaction among warfarin-treated patients with atrial fibrillation, as evidenced by superior outcomes in the INM group compared to the usual care group. This study highlights the potential of integrating WeChat and cloud-based platforms into chronic disease management, particularly during public health crises such as the COVID-19 pandemic. However, given the single-center design, relatively short follow-up period, and reliance on internet access, these findings should be interpreted with caution. The INM should be considered a promising pilot intervention rather than a fully scalable model at this stage. Future research should focus on larger, multicenter trials with extended follow-up durations (e.g., 2–5 years) to assess long-term safety, including bleeding risks and mortality rates, and to evaluate cost-effectiveness across diverse populations.

## Data Availability

The original contributions presented in the study are included in the article/Supplementary Material, further inquiries can be directed to the corresponding author.
